# Supplementary material for: Effect of Oral Antidiabetic Drugs on Tuberculosis Risk and Treatment Outcomes: Systematic Review and Meta-Analysis
Source: Trop Med Infect Dis. 2022 Oct 31;7(11):343. doi: 10.3390/tropicalmed7110343 (PMC9694577; doi:10.3390/tropicalmed7110343)
Supplement: Supplementary file 1 [file tropicalmed-07-00343-s001.zip › tropicalmed-1960835-supplementary.pdf]

Tropical Medicine and Infectious Disease

MDPI

Supplementary Materials: Effect of Oral Antidiabetic Drugs on Tuberculosis Risk and Treatment Outcomes: Systematic Review and Meta-Analysis

Table S1. Search strategy.

	**1. MEDLINE VIA PUBMED**	**Results**	**Date**
#1	"Tuberculosis"[Mesh]	203,082	
#2	(("Sulfonylurea Compounds"[Mesh]) OR ("Thiazolidinediones"[Mesh]) OR ("Glycoside Hydrolase Inhibitors"[Mesh]) OR ("Dipeptidyl-Peptidase IV Inhibitors"[Mesh]) OR ("Sodium-Glucose Transporter 2 Inhibitors"[Mesh]) OR ("meglitinide" [Supplementary Concept]))	43,251	31/August/2022
#3	#1 AND #2	37	
	**2. GOOGLE SCHOLAR**	**Results**	**Date**
#1	All in title "tuberculosis"	718,000	
#2	All in title (("Sulfonylurea") OR ("Thiazolidinediones") OR ("Glycoside Hydrolase Inhibitors") OR ("Dipeptidyl-Peptidase IV Inhibitors") OR ("Sodium-Glucose Transporter 2 Inhibitors") OR ("meglitinide"))	18,500	31/August/2022
#3	#1 AND #2	150	
	**3. SCOPUS**	**Results**	**Date**
#1	TITLE-ABS-KEY (“tuberculosis”)	347,127 31/August/2022 .	
#2	TITLE-ABS-KEY (("Sulfonylurea") OR ("Thiazolidinediones") OR ("Glycoside Hydrolase Inhibitors") OR ("Dipeptidyl-Peptidase IV Inhibitors") OR ("Sodium-Glucose Transporter 2 Inhibitors") OR ("meglitinide"))	58,800	31/August/2022
#3	#1 AND #2	174	
	**4. EMBASE**	**Results**	**Date**
#1	'tuberculosis'/exp	319,200	
#2	'sulfonylurea'/exp OR 'glitazone derivative'/exp OR 'alpha glucosidase inhibitor'/exp OR 'dipeptidyl peptidase IV inhibitor'/exp OR 'sodium glucose cotransporter 2 inhibitor'/exp OR 'meglitinide derivative'/exp	94,649	
#3	#1 AND #2	265	
#4	#3 AND ('case control study'/de OR 'clinical article'/de OR 'clinical trial'/de OR 'clinical trial topic'/de OR 'cohort analysis'/de OR 'comparative effectiveness'/de OR 'comparative study'/de OR 'controlled clinical trial'/de OR 'controlled study'/de OR 'cross sectional study'/de OR 'double blind procedure'/de OR 'drug surveillance program'/de OR 'human'/de OR 'longitudinal study'/de OR 'major clinical study'/de OR 'meta analysis'/de OR 'multicenter study'/de OR 'multicenter study topic'/de OR 'observational study'/de OR 'open study'/de OR 'phase 1 clinical trial topic'/de OR 'phase 2 clinical trial topic'/de OR 'phase 3 clinical trial'/de OR 'phase 3 clinical trial topic'/de OR 'phase 4 clinical trial topic'/de OR 'postmarketing surveillance'/de OR 'prospective study'/de OR 'randomized controlled trial'/de OR 'randomized controlled trial topic'/de OR 'retrospective study'/de OR 'systematic review'/de)	87	31/August/2022
	**5. WEB OF SCIENCE**	**Results**	**Date**
#1	'tuberculosis' (Title)	84,989	
#2	'sulfonylurea' OR ('glitazones derivative' OR 'alpha glucosidase inhibitor' OR 'dipeptidyl peptidase IV inhibitor' OR 'sodium glucose cotransporter 2 inhibitor' OR 'meglitinides') (Title)	5,665	31/August/2022
#3	#1 AND #2	3	
	**6. COCHRANE LIBRARY**	**Results**	**Date**
#1	'tuberculosis' in Title Abstract Keyword	7,175	31/August/2022

#2	Trials matching #1	7,175
#3	'sulfonylurea' OR 'glitazones derivative' OR 'alpha glucosidase inhibitor' OR 'dipeptidyl peptidase IV inhibitor' OR 'sodium glucose cotransporter 2 inhibitor' OR 'meglitinides' in Title Abstract Keyword	5,087
#4	Trials matching #3	5066
#5	#2 AND #4	2
#6	Trials matching #5	2

Table S2. Excluded primary studies.

Study	Reason for Exclusion.
Magee MJ, Salindri AD, Kornfeld H, Singhal A. Reduced prevalence of latent tuberculosis infection in diabetes patients using metformin and statins. Eur Respir J. 2019 Mar 14;53(3):1801695. doi: 10.1183/13993003.01695-2018.	No adjustment for confounders
Singhal A, Jie L, Kumar P, Hong GS, Leow MK, Paleja B, Tsenova L, Kurepina N, Chen J, Zolezzi F, Kreiswirth B, Poidinger M, Chee C, Kaplan G, Wang YT, De Libero G. Metformin as adjunct antituberculosis therapy. Sci Transl Med. 2014 Nov 19;6(263):263ra159. doi: 10.1126/scitranslmed.	No adjustment for confounders
Marupuru S, Senapati P, Pathadka S, Miraj SS, Unnikrishnan MK, Manu MK. Protective effect of metformin against tuberculosis infections in diabetic patients: an observational study of south Indian tertiary healthcare facility. Braz J Infect Dis. 2017 May-Jun;21(3):312-316. doi: 10.1016/j.bjid.2017.01.001.	No adjustment for confounders
Ma Y, Pang Y, Shu W, Liu YH, Ge QP, Du J, Li L, Gao WW. Metformin reduces the relapse rate of tuberculosis patients with diabetes mellitus: experiences from 3-year follow-up. Eur J Clin Microbiol Infect Dis. 2018 Jul;37(7):1259-1263. doi: 10.1007/s10096-018-3242-6.	No adjustment for confounders
Novita BD, Ali M, Pranoto A, Soediono EI, Mertaniasih NM. Metformin induced autophagy in diabetes mellitus - Tuberculosis co-infection patients: A case study. Indian J Tuberc. 2019 Jan;66(1):64-69. doi: 10.1016/j.ijtb.2018.04.003.	No adjustment for confounders.
Leow MK, Dalan R, Chee CB, Earnest A, Chew DE, Tan AW, Kon WY, Jong M, Barkham T, Wang YT. Latent tuberculosis in patients with diabetes mellitus: prevalence, progression and public health implications. Exp Clin Endocrinol Diabetes. 2014 Oct;122(9):528-32. doi: 10.1055/s-0034-1377044.	No adjustment for confounders. The number of exposed and unexposed patients who developed LTBI or did not develop LTBI is not reported.
Kumar NP, Moideen K, Bhootra Y, Nancy A, Viswanathan V, Shruthi BS, Sivakumar S, Natarajan M, Kornfeld H, Babu S. Elevated circulating levels of monocyte activation markers among tuberculosis patients with diabetes co-morbidity. Immunology. 2019 Mar;156(3):249-258. doi: 10.1111/imm.13023.	It does not use clinical outcomes but rather biochemical markers.
Kumar NP, Moideen K, Viswanathan V, Shruthi BS, Sivakumar S, Menon PA, Kornfeld H, Babu S. Elevated levels of matrix metalloproteinases reflect severity and extent of disease in tuberculosis-diabetes co-morbidity and are predominantly reversed following standard anti-tuberculosis or metformin treatment. BMC Infect Dis. 2018 Jul 25;18(1):345. doi: 10.1186/s12879-018-3246-y.	It does not use clinical outcomes but rather biochemical or immunological markers.
Novita BD, Pranoto A, Wuryani, Soediono EI, Mertaniasih NM. A case risk study of lactic acidosis risk by metformin use in type 2 diabetes mellitus tuberculosis coinfection patients. Indian J Tuberc. 2018 Jul;65(3):252-256. doi: 10.1016/j.ijtb.2017.05.008.	It does not use clinical outcomes but rather biochemical markers.
Mishra R, Krishan S, Siddiqui AN, Kapur P, Khayyam KU, Rai PK, Sharma M. Impact of metformin therapy on health-related quality of life outcomes in tuberculosis patients with diabetes mellitus in India: A prospective study. Int J Clin Pract. 2021 Apr;75(4):e13864. doi: 10.1111/ijcp.13864.	It does not use clinical outcomes but rather a multidimensional scale (HRQOL-14).
